# Cardiac autonomic and cortisol stress responses to real operations in surgeons: relationship with individual psychobiological characteristics and experience

**DOI:** 10.1186/s13030-023-00266-5

**Published:** 2023-02-21

**Authors:** Luca Carnevali, Elena Bignami, Sara Gambetta, Margherita Barbetti, Matteo Procopio, Antonio Freyrie, Paolo Carbognani, Luca Ampollini, Andrea Sgoifo

**Affiliations:** 1 Stress Physiology Lab, Department of Chemistry, Life Sciences and Environmental Sustainability, University of Parma, Parma, Italy; 2 Department of Medicine and Surgery, University of Parma, Parma, Italy

**Keywords:** Surgeon, Intraoperative stress, Heart rate variability, Cortisol, Depression

## Abstract

**Background:**

Surgeons are exposed to high levels of intraoperative stress, which could compromise their psychological well-being in the long term. This study aimed at exploring the effects of real operations on the activity of stress response systems (i.e., cardiac autonomic function and hypothalamic–pituitary–adrenal axis) during and in the aftermath of surgery, and the moderating role of individual psychobiological characteristics and different levels of experience (senior vs expert surgeons).

**Methods:**

Heart rate, heart rate variability, and salivary cortisol measures (as indexes of cardiac autonomic and hypothalamic–pituitary–adrenal axis activity, respectively) were assessed during real operations and in the perioperative period in a sample of surgeons (*n* = 16). Surgeons’ psychometric characteristics were collected using questionnaires. Results. Real operations triggered both cardiac autonomic and cortisol stress responses which were independent from surgeons’ level of experience. Intraoperative stress responses did not affect cardiac autonomic activity during the following night but were associated with a blunted cortisol awakening response. Moreover, senior surgeons reported higher levels of negative affectivity and depressive symptoms than expert surgeons prior to the surgery. Lastly, the magnitude of heart rate responses to surgery positively correlated with scores on negative affectivity, depression, perceived stress, and trait anxiety scales.

**Conclusion:**

This exploratory study allows to put forward the hypotheses that in surgeons cardiac autonomic and cortisol stress responses to real operations (i) may be associated with specific individual psychological characteristics regardless of the level of experience, (ii) and may have a longer lasting impact on hypothalamic–pituitary–adrenal axis function with potential implications for surgeons’ physical and psychological well-being.

**Supplementary Information:**

The online version contains supplementary material available at 10.1186/s13030-023-00266-5.

## Background

Surgery is undeniably one of the most stressful professions within and beyond the medical field [5]. Long hours in the operating room, highly intensive procedures, and complex decision making are significant physical and mental sources of intraoperative stress [2, 38]. Evidence indicates that high levels of intraoperative stress may impact surgeons’ performance, particularly in novice surgeons, and compromise patient safety [2, 29, 38]. On the other hand, long-term exposure to interoperative stress without adequate recovery may also affect surgeons themselves and predispose them to burnout and depression [5, 13, 20]. Not surprisingly, reported burnout rates are higher in surgical than non-surgical medical specialties and the general population [4, 12, 13]. Several studies have attempted to objectively measure intraoperative stress in surgeons using stress-related biological parameters, such as heart rate (HR) and heart variability (HRV) as indicators of cardiac autonomic activity and salivary cortisol as a proxy for hypothalamic-pituitary-adrenocortical (HPA) axis activity [33]. Although these studies have provided valuable insight into the cardiac autonomic and HPA axis activation which characterizes intraoperative stress, efforts to investigate this relationship have generally been limited to a small sample size (e.g., [6, 10, 17]), one physiological/biochemical index (e.g., [21, 34]), and surgical simulations which may lack realism in comparison with the real surgical environment [15, 36, 37].

Beside this, two important gaps in the literature can hamper our understanding of the possible relation between long-term exposure to intraoperative stress and high rates of burnout among surgeons. First, the extent to which intraoperative stress impacts on cardiac autonomic and HPA axis function in the aftermath of the surgery. For example, one study divided surgeons on the basis of self-report perceived intraoperative stress and found that those who reported higher stress in the operating room had lower vagally-mediated HRV during the following night [28]. However, the design of this study did not clarify whether decreased HRV at night was indeed a consequence of high intraoperative stress. Further, no studies have investigated the effects of intraoperative stress on daily cortisol values. The second open issue is the extent to which individual psychobiological characteristics moderate the impact of intraoperative stress. In fact, it is known that there are large individual differences in the way people cope and respond to stressful situations, and surgeons are no exception. Research in the field has attempted to link intraoperative cardiac autonomic activation to different levels of experience or self-report measures of state anxiety and perceived stress, providing mixed results [6, 23, 34]. Moreover, coping strategies during surgery have been correlated with surgeons’ intraoperative performance [36], but not with cardiac autonomic or cortisol stress reactivity. Further, no studies have investigated whether individual differences in cardiac autonomic and cortisol responses to real surgeries reflect general responses to stressful scenarios or are specific of the surgical environment.

To start filling these two knowledge gaps, this exploratory study describes cardiac autonomic (HR and HRV) and cortisol responses to real operations as markers of intraoperative stress in a sample of surgeons. To address the former, we evaluated the effects of intraoperative stress on cardiac autonomic (HR and HRV sleep measures) and HPA axis (daily cortisol values) activity in the aftermath of surgery. To address the latter, we investigated whether individual differences in intraoperative stress responses were associated with different levels of experience (senior vs expert surgeons), specific psychological and personality characteristics, coping strategies, and/or cardiac autonomic and cortisol responses to a laboratory stressor (i.e., an adapted version of the Trier Social Stress Test) [22, 26].

## Methods

### Participants

This study was conducted in a sample of surgeons from the “Maggiore Hospital” in Parma (Italy). Eligibility criteria comprised being the primary surgeon of major procedures lasting at least 120 min, absence of current or past psychiatric and cardiac disorders, and body mass index (BMI) < 30 kg/m^2^. The divisions of urology and vascular, thoracic, and general surgery were involved. A sample size calculation was not deemed necessary because of the exploratory nature of this study. Nineteen surgeons consented to participate, with three surgeons terminating participation prior to the conclusion of the study due to non-compliance. The final sample consisted of 16 surgeons (12 males and 4 females; mean ± SD: age = 45.8 ± 11.6 years, experience = 14.1 ± 11.9 years from specialization, BMI 23.5 ± 0.6 kg/m^2^), with specialization in urology (*n* = 2), vascular surgery (*n* = 7), thoracic surgery (*n* = 4), and general surgery (*n* = 3). The median split of years of experience (calculated from the year of specialization) was used to divide the sample in two groups, senior (more than 10 years of experience) and expert (less than 10 years of experience) surgeons. The general characteristics of these two groups are listed in Table 1. The study conformed to the Declaration of Helsinki, and the protocol (Comitato Etico Area Vasta Emilia Nord, prot.n. 9561) was approved by the local Ethical Committee, Italy. All volunteers gave written informed consent to participate. Before the beginning of the operation, patients were informed that two saliva samples were going to be collected from the surgeon (see below) without interrupting or interfering with the surgery.

**Table 1:** General characteristics of senior and expert surgeons

	**Senior (*n* = 8)**	**Expert (*n* = 8)**	**t/χ**^**2**^	**p**
Age (years)	55.3 ± 3.1	36.3 ± 0.8	5.95	< .001
Experience (years)	23.8 ± 3.2	4.4 ± 0.8	5.90	< .001
Females (n)	2	2	0	1
BMI (kg/m^2^)	23.8 ± 0.9	23.2 ± 0.8	.48	.638
Smokers (n)	1	1	0	1
Medical specialty (n)	3V 0G 3T 2U	4V 3G 1T 0U	6.14	.105
Duration of surgery (min)	197.5 ± 24.7	164.8 ± 17.1	1.09	.29

Data are reported as mean ± standard error

*Abbreviations:* *V* Vascular surgery, *G* General surgery, *T* Thoracic surgery, *U* Urology

### Study design

**Fig. 1:**
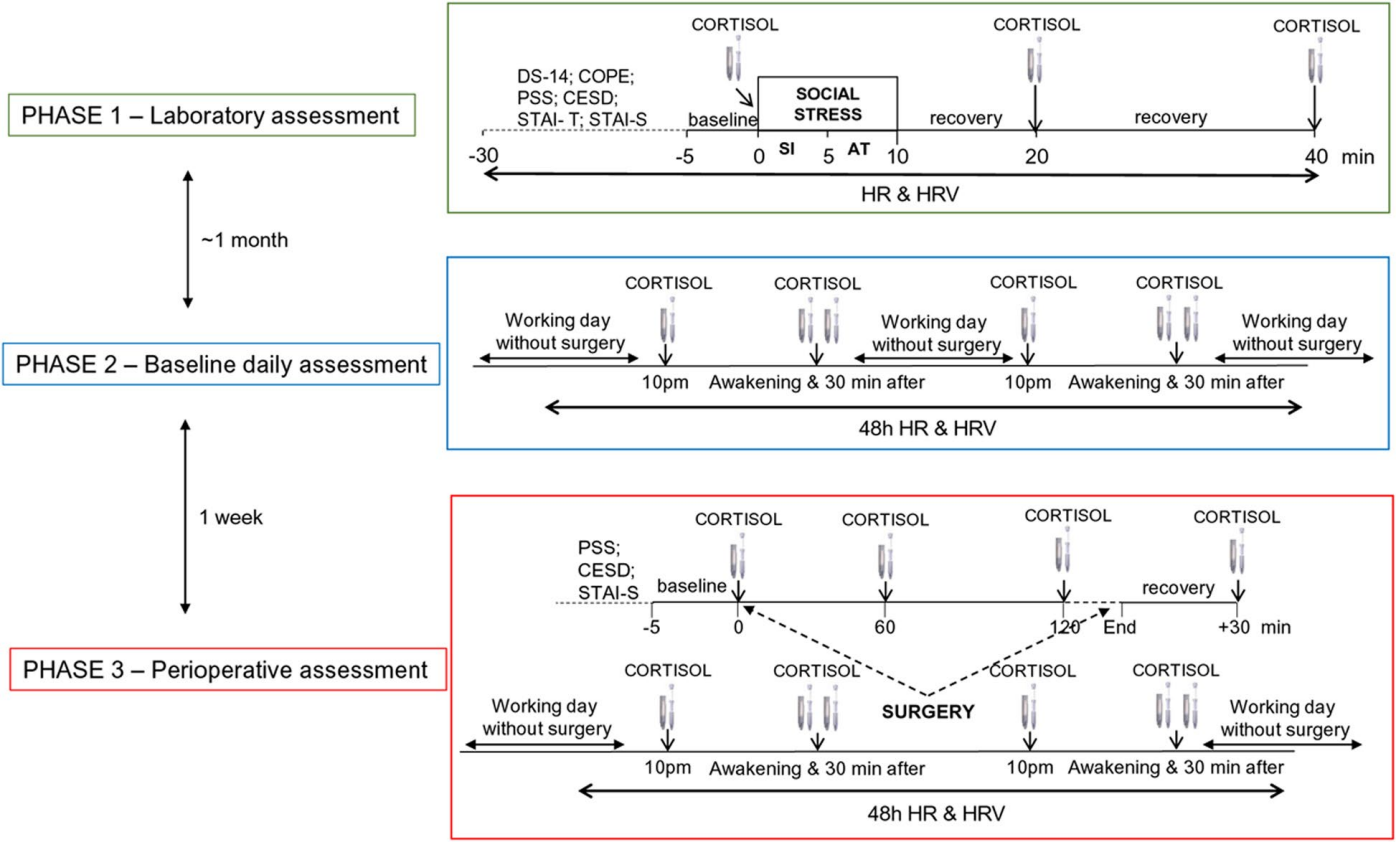
Timeline of the experimental procedures during the three phases of the study protocol. Abbreviations: DS-14 = Type D Personality Scale; COPE = Coping Orientation to Problems Experienced; CESD = Center for Epidemiological Studies Depression Scale; PSS = Perceived Stress Scale; STAI-T = State-Trait Anxiety Inventory, Trait version; STAI-S = State-Trait Anxiety Inventory, State version; SI = stress interview; AT = arithmetic task; HR = heart rate; HRV = heart rate variability

This observational prospective study was conducted over three different phases, namely laboratory assessment, daily baseline assessment, and perioperative assessment (Fig. 1).

#### Laboratory assessment

The top panel of Fig. 1 depicts the sequence of events included in the first phase of the study protocol which took place in a designated room at the “Maggiore Hospital” in Parma between 2 and 5 pm. Surgeons were recommended to refrain from caffeine, alcohol, and nicotine consumption, as well as strenuous exercise for at least 2 h prior to the laboratory assessment, as these variables may have transient effects on cardiovascular/neuroendocrine measurements [24]. Upon arrival, surgeons were fitted with the Firstbeat Bodyguard 2 device (Firstbeat Technologies, Finland) for recordings of R-R intervals. Subsequently, they were allowed to settle down in the new environment for 30 min while sitting on a comfortable chair in front of two familiar experimenters. During this adaptation period, they completed a series of socio-demographic, lifestyle, and dispositional scales (see below “Psychometric questionnaires”). After baseline R-R interval recordings, a saliva sample was collected from each surgeon using oral swabs and swab storage tubes (Salimetrics, Cambridge, UK). Subsequently, surgeons were submitted to a social stress test which was based on an adapted version of the Trier Social Stress Test [22, 26]. During the 5-min stress interview (SI) phase, they were asked to answer a series of questions about how they behave and feel in different social contexts. Subsequently, they were asked to complete a 5-min mental arithmetic task (AT) by counting aloud backwards from 2083 by 13’s. The SI and AT were administered by an unfamiliar interviewer of the opposite sex, with a small unfamiliar audience (two people) sitting behind the surgeons. Upon completion of the stress phases, surgeons remained seated and quiet in the presence of the two familiar experimenters for the following 30-min recovery phase. Additional saliva samples were collected 20 and 40 min after the beginning of the SI.

#### Baseline daily assessment

The middle panel of Fig. 1 depicts the sequence of events included in the second phase of the study protocol. Approximately one month after the laboratory session, surgeons were summoned to the designated room at the “Maggiore Hospital” in Parma during a working day in which they did not perform any surgery. Of note, they had not performed any surgery also on the preceding day. Initially, they were fitted with the Firstbeat Bodyguard 2 device and instructed to wear it for the following 48 h over working days without their involvement in any surgical procedure. Furthermore, they received oral and written instructions on how to collect and store saliva samples at home during the same 48 h. Specifically, saliva samples were collected at 10 pm, immediately after awakening, and exactly 30 min after awakening, using oral swabs and clearly marked swab storage tubes (Salimetrics, Cambridge, UK). Finally, they were given diaries for self-reporting of bedtime, wake time, and time of saliva sample collection.

#### Perioperative assessment

The bottom panel of Fig. 1 depicts the sequence of events included in the third phase of the study protocol. The week after the baseline assessment, surgeons were summoned to the designated room at the “Maggiore Hospital” in Parma the day before their involvement as primary operators in a major surgical procedure with an estimated duration of at least 120 min. Of note, surgeons did not perform any surgery during that day nor the day before. Like the previous phase, they were asked to wear the Firstbeat Bodyguard 2 device and to collect and store saliva samples at home during the following 48 h (i.e., at 10 pm, immediately after awakening, and exactly 30 min after awakening) and to self-report bedtime, wake time, and time of saliva sample collection on their diaries. On the day of the surgery, they were recommended to refrain from caffeine, alcohol, and nicotine consumption, as well as strenuous exercise for at least 2 h prior to the surgical procedure which started between 9 and 12 am. Upon their arrival at the operating room, surgeons completed a series of psychometric questionnaires while sitting on a comfortable chair (Fig. 1, bottom panel, inner box). After 5 min of baseline R-R interval recordings, a saliva sample was collected from each surgeon. Then, surgeons got ready for the surgical procedure which began within the following 15 min. The start of the surgery was standardized for each surgeon as the moment in which the first surgical incision was made. Saliva samples were collected 1 h and 2 h after the beginning of the surgical procedure by passing the oral swab behind the surgeons’ mask and without interrupting the operation. Upon its completion, surgeons were allowed a 30-min recovery period outside the operating room, at the end of which and an additional saliva sample was collected (Fig. 1, bottom panel, inner box). Then, surgeons were de-briefed and continued with the daily R-R interval recordings and saliva sample collection as instructed.

### Psychometric questionnaires

The Italian version of the Type D Personality Scale (DS14), which consists of two 7‐item subscales, was used to assess the presence of the personality traits of negative affectivity (e.g., “I am often irritated”) and social inhibition (e.g., “I find it hard to start a conversation”) [11]. Each item is rated on a 5‐point Likert‐type scale ranging from 0 to 4. Both subscales show high internal consistency (Cronbach's α 0.82 and 0.80) and reliability (*r* = 0.62 and 0.81). The DS-14 was administered once during phase 1 (Fig. 1).

Coping strategies were assessed using the Italian version of the Coping Orientation to Problems Experienced questionnaire (COPE‐NVI; [30]), which is a 60‐item, self‐report instrument that evaluates five independent dimensions of coping: social support, avoidance strategies, positive attitude, task‐oriented, and transcendental orientation. All items are rated on a 4‐point scale: 1 (I usually don't do this at all); 2 (I usually do this a little bit); 3 (I usually do this a medium amount); 4 (I usually do this a lot). The COPE‐NVI shows high internal consistency (Cronbach's α from 0.62 to 0.92) and reliability (coefficients ranging from 0.48 to 0.77; [30]). The COPE-NVI was administered once during phase 1 (Fig. 1).

The severity of trait anxiety was measured using the trait version of the State-Trait Anxiety Inventory (STAI) [31], which is a 4‐point Likert scale consisting of 20 items assessing how the subject feels, independent from the status and circumstances (e.g., “I feel secure,” I feel troubled”). The lowest score that can be obtained is 20 and the highest is 80. Higher scores indicate higher anxiety levels. The validity of this scale has been repeatedly confirmed, with reliability coefficients ranging from 0.71 to 0.86 and internal consistency and homogeneity coefficients between 0.83 and 0.87. State anxiety was measured using the state version of the STAI, which asks how respondents feel “right now” using 4‐point Likert scale items that measure subjective feelings of apprehension, tension, nervousness, worry, and activation/arousal of the autonomic nervous system. The reliability coefficient is 0.62. The trait version of the STAI was administered once during phase 1, whereas the state version was administered during both phase 1 and 3 (Fig. 1).

The Perceived Stress Scale (PSS) is the most widely used psychological instrument for measuring the perception of stress. It is a measure of the extent to which situations in one’s life are appraised as stressful [9]. Items were designed to tap how unpredictable, uncontrollable, and overloaded respondents find their lives. The 10 questions that compose this test are related to feelings and thoughts experienced during the last month. In each case, respondents are asked how often they felt a certain way. Scores ranging from 14 to 26 are considered “moderate perceived stress”, those ranging from 27 to 40 are considered “high perceived stress” [9]. The PSS was administered twice, during phase 1 and 3 (Fig. 1).

The Center for Epidemiological Studies Depression Scale (CES-D) is a 20-item self-report scale designed to measure depressive symptomatology during the past week in the general population [27]. The total score ranges from 0 to 60. Standard cutoffs are > 16 for mild depression and > 23 for clinical depression. Cronbach’s alphas are above 0.85 in the general population and 0.90 in patients with depression confirming high reliability [27]. The CES-D was administered twice, during phase 1 and 3 (Fig. 1).

### Heart rate and heart rate variability analysis

Raw R-R intervals obtained with the Firstbeat Bodyguard 2 device were arranged in 5-min epochs and analyzed with the Kubios HRV software [32], using a medium filter for threshold-based artefact correction [1]. For each epoch, separate estimates of HR (reported in beats per minute) and HRV were generated. The root mean square of successive beat-to-beat interval differences (RMSSD, ms) was considered as a vagally-mediated index of HRV [24]. Specific analyses are detailed below.

#### HR and HRV responses to the social stress test

Average HR and HRV values were calculated for each 5-min epoch. Also, delta HR and HRV values were obtained for each subject by subtracting the baseline value from the respective HR and HRV values during the SI and AT phases.

#### HR and HRV responses to surgery

Surgeries lasted at least 120 min (range 120–365 min). Therefore, to standardize the analysis, HR and HRV data from each 5-min epoch during surgery were further averaged to obtain mean HR and HRV values during the first and second hour of the surgical procedure. Delta HR and HRV values were obtained for each subject by subtracting the baseline values from the respective average HR and HRV values during the first two hours of surgery.

#### HR and HRV during sleep

HR and HRV data from each 5-min epoch were further averaged for sleep hours based on self-reports of bed and wake up times.

### Cortisol analysis

To collect saliva, participants were asked to keep oral swabs under the tongue for 2 min. Immediately after collection, saliva samples were frozen at − 20 °C. For the analysis, samples were thawed, brought to room temperature and centrifuged (1500 g × 10 min), resulting in a clear supernatant of low viscosity. Salivary cortisol levels were determined by enzyme-linked immunosorbent assay (High Sensitivity Salivary Cortisol Enzyme Immunoassay Kit: Salimetrics LLC, State College, PA) by a researcher who was blind to the design of the experiment. Samples were assayed in duplicates following kit instructions with a 96-well plate, using the BioTek 800 TS absorbance reader and Gen5 software (BioTek Instruments Inc., Vermont, USA). To avoid inter-assay variability all samples from the same participant were assayed in the same batch. The inter-assay and intra-assay coefficients of variability were 5.9 and 8.4, respectively. Specific analyses are detailed below.

#### Cortisol responses to the social stress test

Initially, mean salivary cortisol values were obtained for each collection point. Delta cortisol values were then calculated by subtracting the baseline value from the cortisol values obtained after stress exposure.

#### Cortisol responses to surgery

Initially, mean salivary cortisol values were calculated for each collection point. Delta cortisol values were then obtained by subtracting the baseline value from the cortisol value obtained after 1 h of surgery.

#### Daily cortisol levels

Initially, mean salivary cortisol values were calculated for each collection point. The cortisol awakening response (CAR) was calculated by subtracting cortisol values obtained 30 min after awakening from salivary cortisol values obtained immediately after awakening. CAR values during the first and second day of baseline assessment were further averaged to obtain baseline CAR values.

### Statistical analysis

Data are expressed as means ± standard error (SE). Statistical analyses were performed with the software package SPSS (version 28) (SPSS Inc., Chicago, IL). Statistical significance was set at *p* < 0.05. The normal distribution of variables was determined using the Kolmogorov–Smirnov test.

Differences between the two groups (senior vs expert) in age, years of experience, number of females and smokers, medical specialty and duration of surgery, and psychometric characteristics were analyzed by Student's t-tests and χ2 tests.

Cardiac autonomic and cortisol responses to the social stress test and surgery were analyzed with a series two-way ANOVAs for repeated measures, with “group” (senior vs expert) as the between subject factor and “recording period” as the within-subject factor.

Daily cortisol levels were analyzed by a one-way ANOVA for repeated measures, with “time” (3 levels: 10 pm; awakening and 30 min after awakening) and “day of assessment” (4 levels: baseline 1, baseline 2, pre-surgery, post-surgery) as the within-subject factors. CAR values and HR and HRV sleep values were analyzed with a series of two-way ANOVAs for repeated measures, with “group” (senior vs expert) as the between subject factor and “day of assessment” as the within-subject factor. Pre-planned analyses were conducted using Student's t-tests, with a Bonferroni correction for multiple comparisons for each outcome variable separately.

Partial correlation analyses (controlling for sex and level of experience) were computed to compare: (i) HR, HRV and cortisol responses to social stress with those observed in responses to surgery, (ii) psychometric characteristics to HR, HRV and cortisol responses to social stress, and (iii) psychometric characteristics to HR, HRV and cortisol responses to surgery.

## Results

### General characteristics

The general characteristics of senior and expert surgeons who participated in the study are reported in Table 1. Importantly, there were no differences in the proportion of women and smokers between the two groups, neither in the distribution of surgeons in different medical specialties nor in the duration of the surgery. The different types of surgery and their specific duration are listed in Table 2.

**Table 2:** Type and duration of surgeries

Surgeon	Specialty	Type of surgery	Duration (min)
S01	Vascular surgery	Abdominal aortic aneurysms (open access)	165
S02	Vascular surgery	Carotid endarterectomy	150
S03	Vascular surgery	Percutaneous Transluminal Angioplasty of Femoral Artery	120
S04	Vascular surgery	Endovascular aortic repair	155
S05	Thoracic surgery	Pneumonectomy	180
S06	Vascular surgery	Endovascular aortic repair + femoral endarterectomy	200
S07	Thoracic surgery	Robotic Lobectomy	365
S08	Thoracic surgery	Lung segmentectomy	215
S09	Vascular surgery	Carotid endarterectomy	123
S10	General surgery	Sleeve gastrectomy	140
S11	Thoracic surgery	Dual Lobectomy in VATS	190
S15	General surgery	Treatment of Laparocele with prothesis	155
S16	General surgery	Gastric band removal	121
S17	Urology	Nephroureterectomy (open access)	150
S18	Urology	Nephroureterectomy (open access)	180
S19	Vascular surgery	Femoral Popliteal Bypass Surgery	260

### Psychometric characteristics

Table 3 shows psychometric characteristics of senior and expert surgeons. Senior surgeons reported significantly higher scores on the negative affectivity subscale of the DS-14, with a large effect size. Similarly, senior surgeons reported significantly higher depressive symptoms at both assessment points, with a large effect size in both instances. Of note, depressive symptoms prior to the social stress test significantly correlated with depressive symptoms prior to the surgery (*r* = 0.856, *p* < 0.001). In addition, senior surgeons were 1 standard deviation higher in trait anxiety levels, although this difference did not reach full statistical significance. Perceived stress levels prior to the social stress test significantly correlated with perceived stress levels prior to the surgery (*r* = 0.768, *p* < 0.001). Of note, senior surgeons reported nearly 0.7 and 0.9 standard deviation higher levels of stress in both instances, respectively. No group differences were found for coping strategies and state anxiety levels prior to the social stress test and surgery.

**Table 3:** Psychometric characteristics in senior and expert surgeons

	**Senior (*n* = 8)**	**Expert (*n* = 8)**	**t**	**p**	**Effect size (Cohen’s d)**
*DS‐14 (score)*					
Negative affectivity	11.3 ± 1.6	5.5 ± 1.3	2.76	.015	1.38
Social inhibition	8.9 ± 2.1	9.1 ± 1.6	.09	.927	.05
*COPE (score)*					
Social support	29.6 ± 1.7	32.1 ± 2.6	.80	.436	.40
Avoidance	21.8 ± 1.1	20.9 ± 2.2	.36	.722	.18
Positive attitude	32.4 ± 1.5	33.6 ± 1.4	.60	.560	.30
Task oriented	32.4 ± 1.6	34.8 ± 1.9	.95	.360	.47
Transcendent orientation	21.6 ± 1.8	21.3 ± 1.8	.14	.887	.07
*CESD (score)*					
Depressive symptoms (pre-social stress test)	15.5 ± 2.9	7.8 ± 0.9	2.50	.025	1.25
Depressive symptoms (pre-surgery)	18.4 ± 2.5	10.0 ± 1.5	2.84	.013	1.42
*PSS (score)*					
Perceived stress (pre-social stress test)	17.3 ± 2.2	13.2 ± 1.9	1.36	.195	.68
Perceived stress (pre-surgery)	19.6 ± 2.0	14.8 ± 1.9	1.80	.093	.90
*STAI (score)*					
Trait anxiety	39.8 ± 2.6	33.0 ± 1.9	2.10	.054	1.05
State anxiety (pre-social stress)	36.9 ± 8.7	33.0 ± 1.9	.51	.617	.26
State anxiety (pre-surgery)	37.9 ± 2.5	35.1 ± 1.4	.94	.361	.47

Data are reported as mean ± standard error

*Abbreviations:* *DS-14* Type D personality scale, *COPE* Coping orientation to problems experienced, *CESD* Center for epidemiological studies depression scale, *PSS* Perceived stress scale, *STAI* State-trait anxiety inventory

### Cardiac autonomic and cortisol responses to the social stress test

**Fig. 2:**
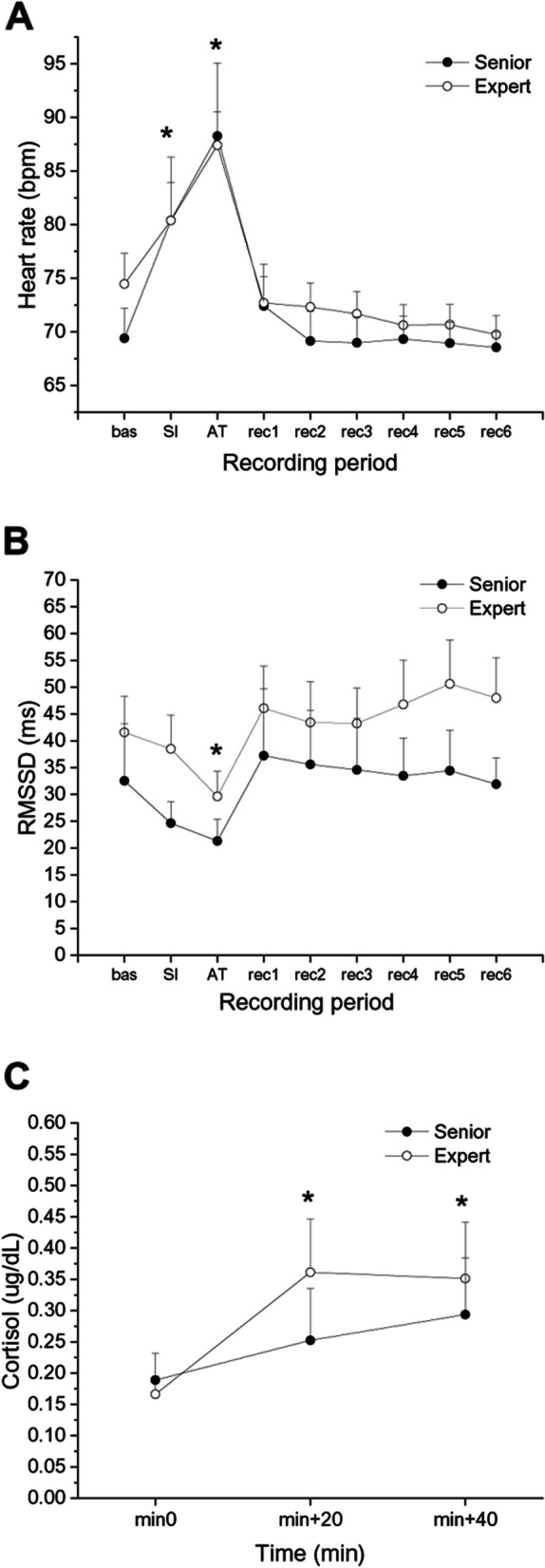
Heart rate (**A**), heart rate variability (**B**), and cortisol (**C**) responses to the social stress test in senior and expert surgeons (*n* = 8 per group). Data are reported as mean ± standard error. Abbreviations: bas = baseline; SI = social interview; AT = arithmetic task; rec = recovery; RMSSD = root mean square of successive beat-to-beat interval differences. * *p* < .05 vs baseline/min 0 value for both groups

Two-way ANOVAs for repeated measured yielded a significant effect of “recording period” for HR (F = 23.25, *p* < 0.001, η_p_^2^ = 0.624), RMSSD (F = 4.62, *p* < 0.001, η_p_^2^ = 0.268) and cortisol (F = 6.64, *p* < 0.01, η_p_^2^ = 0.322) values during the social stress test. Considering the full sample of surgeons, HR was significantly higher both during the SI (80.4 ± 3.4 bpm, *p* < 0.01) and the AT (87.8 ± 3.7 bpm, *p* < 0.001) compared with the mean baseline value (71.9 ± 2.0 bpm). RMSSD values were significantly lower only during the AT (25.5 ± 3.1 ms, *p* < 0.05) compared with the mean baseline value (37.1 ± 6.3 ms). These effects were independent from surgeons’ experience (Fig. 2A and B). Likewise, cortisol values in the full sample were significantly higher both 20 min (0.307 ± 0.059 µg/dL, *p* < 0.05) and 40 min after (0.323 ± 0.064 µg/dL, *p* < 0.05) stress onset compared with the mean baseline value (0.178 ± 0.026 µg/dL, *p* < 0.05), with no differences between senior and expert surgeons (Fig. 2C).

### Cardiac autonomic and cortisol responses to surgery

**Fig. 3:**
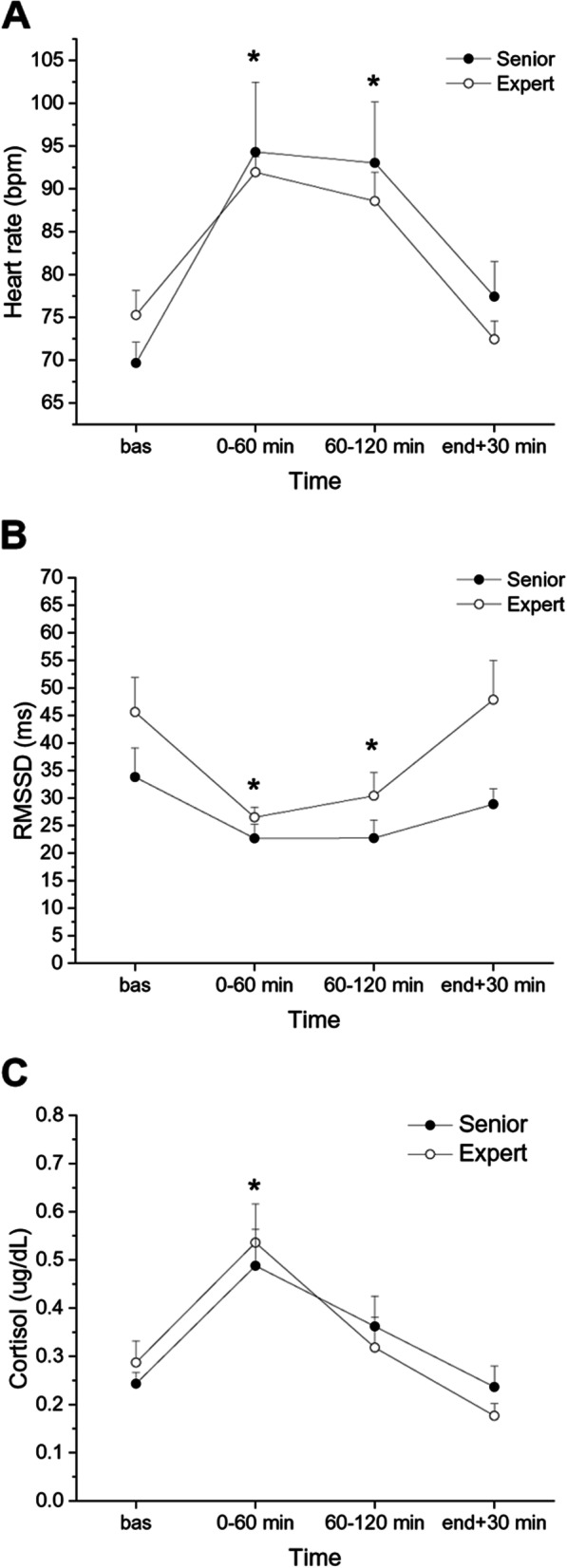
Heart rate (**A**), heart rate variability (**B**), and cortisol (**C**) responses to surgery in senior and expert surgeons (*n* = 8 per group). Data are reported as mean ± standard error. Abbreviation: RMSSD = root mean square of successive beat-to-beat interval differences. * *p* < .05 vs baseline value for both groups

Two-way ANOVAs for repeated measured yielded a significant effect of “recording period” for HR (F = 20.76, *p* < 0.001, η_p_^2^ = 0.597), RMSSD (F = 13.05, *p* < 0.001, η_p_^2^ = 0.482) and cortisol (F = 16.25, *p* < 0.001, η_p_^2^ = 0.537) values during surgery. Considering the full sample of surgeons, HR was significantly higher both during the first (91.1 ± 4.2 bpm, *p* < 0.001) and second (90.8 ± 3.9 bpm, *p* < 0.001) hour of surgery compared with the mean baseline value (72.5 ± 1.9 bpm). RMSSD values were significantly lower both during the first (24.6 ± 1.6 ms, *p* < 0.001) and second hour (26.6 ± 2.7 ms, *p* < 0.001) compared with the mean baseline value (39.7 ± 4.1 ms). Importantly, these effects were independent from surgeons’ experience (Fig. 3A and B).

Moreover, cortisol values in the full sample were significantly higher only after the first hour of surgery (0.512 ± 0.055 µg/dL, *p* < 0.001) compared with the mean baseline value (0.265 ± 0.025 µg/dL, *p* < 0.05), with no differences between senior and expert surgeons (Fig. 3C).

### Correlations between cardiac autonomic and cortisol stress responses and psychometric characteristics

No significant correlations were found between HR, RMSSD, and cortisol responses to social stress and surgery (Table S1, Supplementary material). On the other hand, we found significant positive correlations between HR responses to surgery (i.e., HR delta values) and negative affectivity, trait but not state anxiety (Table 4), depressive symptoms and perceived stress, controlling for sex and experience. No significant correlations were found between psychometric variables and RMSSD and cortisol responses to surgery (Tables S2, Supplementary material). Likewise, no significant correlations were found between psychometric characteristics and HR, RMSSD and cortisol responses to the social stress test (Table S3, Supplementary material).

**Table 4:** Partial correlations (controlling for sex and experience) between psychometric characteristics and heart rate responses to surgery for the full sample of surgeons (*n* = 16)

		1	2	3	4	5	6
1. DS-14 (NA)	r	-					
	p	-					
2. STAI-T	r	.738	-				
	p	< .01	-				
3. STAI-S	r	.422	.248	-			
	p	.133	.392	-			
4. CESD	r	.509	.798	.430	-		
	p	.063	< .001	.125	-		
5. PSS	r	.471	.509	.229	.618	-	
	p	.089	.063	.431	.018	-	
6. Delta HR	r	.638	.804	-.007	.568	.575	-
	p	.014	< .001	.981	.034	.031	-

Only significant correlations between delta heart rate (HR) responses to surgery and psychometric characteristics are reported

*Abbreviations:* *DS-14 (NA)* Type D personality scale, negative affectivity subscale, *STAI-T* State-Trait anxiety inventory, Trait version, *STAI-S* State-trait anxiety inventory, state version, *CESD* Center for epidemiological studies depression scale, *PSS* Perceived stress scale

### Daily cortisol values

**Fig. 4:**
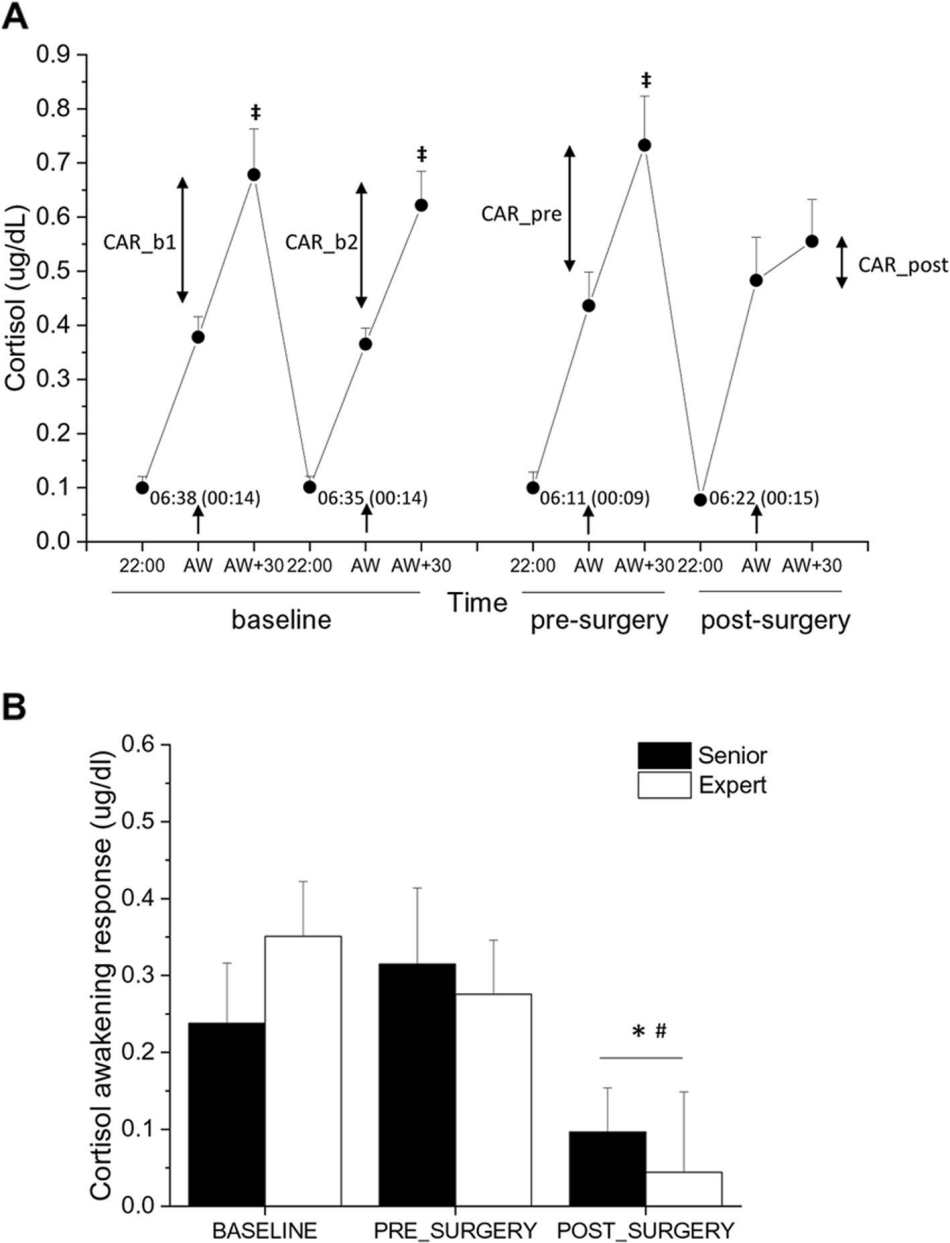
Panel A depicts cortisol values at 22:00 h, immediately upon awakening (AW), and 30 min after awakening (AW + 30) in the full sample of surgeons (*n* = 16) across the different daily phases. Abbreviation: CAR = cortisol awakening response. Inner numbers report the average self-reported time (h:min) of awakening. Data are reported as mean ± standard error. ^‡^ = *p* < .05 vs the respective awakening value. Panel B represents CAR values in baseline and pre- and post-surgery conditions in senior and expert surgeons (n = 8 per group). Data are reported as mean ± standard error. * *p* < .05 vs baseline value and ^#^ *p* < .05 vs pre-surgery value for both groups

One-way ANOVA for repeated measures yielded a significant “time x day of assessment” interaction on daily cortisol levels during the baseline and perioperative assessments (F = 2.84, *p* < 0.05, η_p_^2^ = 0.168) (Fig. 4A). Specifically, cortisol values 30 min after awakening were significantly higher compared with the values upon awakening both in baseline condition and the day of the surgery (*p* < 0.05), but not the day after the surgery (Fig. 4A), suggesting a flattening of the CAR. In fact, two-way ANOVA for repeated measures yielded a significant effect of “day of assessment” on CAR values (F = 6.84, *p* < 0.01, η_p_^2^ = 0.345). Considering the full sample of surgeons, CAR values were significantly lower during the post-surgery assessment (0.070 ± 0.060 µg/dL) compared with the pre-surgery (0.295 ± 0.064 µg/dL, *p* < 0.01) and baseline (0.281 ± 0.058 µg/dL, *p* < 0.05) assessments. This effect was independent from surgeons’ experience (Fig. 4B). Of note, no differences were found in average wake-up times across the different assessment phases (Fig. 4A).

### HR and HRV sleep values

There were no differences in HR and RMSSD sleep values at the three assessment points in the full sample of surgeons (HR: baseline = 59.0 ± 1.8 bpm, pre-surgery = 58.7 ± 1.8 bpm, post-surgery = 60.0 ± 2.5 bpm; RMSSD: baseline = 39.3 ± 4.4 ms; pre-surgery = 42.2 ± 2.9 ms; post-surgery = 40.3 ± 3.4 ms) and between the two groups (senior vs expert) (Table 5).

**Table 5:** Heart rate and heart rate variability sleep values in senior (*n* = 8) and expert (*n* = 8) surgeons

		baseline	pre-surgery	post-surgery
HR (bpm)	Senior	56.7 ± 2.1	59.2 ± 2.9	62.0 ± 4.4
	Expert	61.5 ± 3.2	58.4 ± 2.6	58.3 ± 2.6
RMSSD (ms)	Senior	40.8 ± 7.6	42.0 ± 5.2	35.7 ± 5.6
	Expert	37.4 ± 5.0	42.4 ± 3.5	44.2 ± 3.4

Data are reported as mean ± standard error. Abbreviations: *HR* Heart rate, *RMSSD* Root mean square of successive beat-to-beat interval differences

## Discussion

To the best of our knowledge, this is the first study to combine the assessment of both cardiac autonomic (HR and HRV) and cortisol parameters as markers of intraoperative stress in surgeons during real operations. Findings of increased HR and reduced vagally-mediated HRV during the first two hours of surgery are indicative of cardiac sympathetic activation/vagal withdrawal and complement previous results obtained in smaller samples (e.g., [17, 23, 34]) or during surgical simulations (e.g., [36, 37]). Further, the described increase in salivary cortisol levels after the first hour of surgery is indicative of HPA axis activation and represents an additional objective characterization of intraoperative stress response. Cortisol levels after two hours of surgery were no longer different from the baseline level, a finding that somewhat replicates the time course of cortisol changes described in a smaller sample of surgeons (*n* = 7) during complex laparoscopic procedures [14]. Of note, a previous study did not find any increases in salivary cortisol levels in surgeons during surgical simulations [36]. The authors of this study argued that surgical stress – in real life or as a simulation – may not influence HPA axis activity because the activity of this axis would be more sensitive to social stressors and elements of low control [36]. Our findings point to a slightly different interpretation. Specifically, we argue that elements of low control may lack in surgical simulations, but not in the real surgical environment where technical problems and equipment failure may jeopardize patient safety and, most importantly, patient complication may occur. Therefore, surgical simulations may be sub-optimal for studying intraoperative stress responses.

Moreover, one might expect that younger surgeons experience more intraoperative stress than senior surgeons who are used to managing intraoperative complications and responsibility. In our study, this hypothesis could not be confirmed. Indeed, both subjective (STAI state scores) and objective (HR, HRV, and cortisol responses) indicators of stress were similar between senior and expert surgeons. We conclude that real operations are a significant source of stress for surgeons and trigger both cardiac sympathetic and HPA axis activation which cannot be moderated by professional experience.

### Effects of intraoperative stress on cardiac autonomic and HPA axis function after surgery

A largely unexplored issue in this field of research is the impact of intraoperative stress on cardiac autonomic and HPA axis function in the aftermath of surgery. Here, we found that 30 min after the end of surgery, both cardiac autonomic parameters and cortisol values returned to the respective pre-surgery levels, suggesting complete recovery.

Likewise, HR and HRV sleep values during the night after surgery did not differ from the previous night (pre-surgery) and from the baseline condition, suggesting that cardiac autonomic function at night is not affected by intraoperative stress. This is relevant because nighttime HR and HRV values have prognostic value across a range of established cardiovascular risk factors in both diseased and apparently healthy individuals [18, 19]. Of note, a previous study divided surgeons into a stressed and a non-stressed group based on the difference in STAI scores from pre- to postoperative rating. The authors found that those who reported higher intraoperative stress (i.e., increases in STAI scores) had lower HRV, but no changes in HR, during the following night [28]. However, this study did not include a pre-surgery and/or baseline assessment of HR and HRV night values and therefore did not allow to clarify whether decreased HRV at night was a longer lasting effect of intraoperative stress or was a characteristic of that sub-group of surgeons. Unfortunately, in the current study STAI scores were not rated postoperatively and therefore we could not replicate/confute the results obtained by Rieger and colleagues using their approach [28].

On the other hand, we found a blunted cortisol awakening response (CAR) the morning after surgery compared with both the pre-surgery and the baseline assessment, which suggests that intraoperative stress may have a longer lasting impact on HPA axis function. Notably, this effect was comparable between senior and expert surgeons. While intraoperative stress seemed to have no effects on evening cortisol values, a blunted CAR was also described in healthy individuals the morning after acute mental/social stress exposure [7, 16]. Deviations from the typical increase in cortisol following awakening are characteristic of HPA axis dysregulation and associated with negative consequences for health and well-being. For example, blunted CARs are associated with fatigue, burnout, and exhaustion [8]. Therefore, our results suggest that surgeons may still be recovering from intraoperative stress the next morning as reflected by a failure to fully activate the HPA axis after awakening. Our interpretation is strengthened by the fact (i) baseline CAR was assessed on two consecutive working days to increase the reliability of this measure and was similar to the pre-surgery value, (ii) wake-up hours were similar across the different assessment phases, and (iii) statistical significance survived when the duration of the surgery was entered as a covariate. Nevertheless, this new finding asks for a more elaborate replication, for example with an extension of cortisol sampling days to verify whether a blunted CAR characterizes only the morning after surgery or persists for more days. This is particularly relevant because there is evidence for a blunted CAR in both clinical and non-clinical burnout [25] and a recent meta-analysis reported a high prevalence of burnout among surgeons from different countries and medical specialties [20]. Therefore, the presence of a blunted CAR after surgery suggests a potential pathway by which repeated exposure to intraoperative stress might ultimately predispose vulnerable surgeons to burnout.

### Associations between psychobiological characteristics and intraoperative stress responses

In this study, senior surgeons reported higher levels of negative affectivity, depressive symptoms, and trait anxiety compared to expert surgeons. Importantly, depressive scores in senior surgeons were around the standard cut-off for mild depression. This could be attributed to the fact that more experienced surgeons are confronted with many other work stressors, such as higher responsibility and administrative and teaching duties, which may have an influence on their subjective perception of stress and psychological well-being. However, as discussed above, we cannot rule out the possibility that a longer history of repeated exposure to intraoperative stress may ultimately have a negative impact on the psychological well-being of more experienced surgeons. Interesting, from a dimensional perspective, depressive symptoms, negative affectivity, trait anxiety, and perceived stress strongly and positively correlated with the magnitude of HR responses to real operations. Remarkably, similar associations between psychological characteristics and HR stress responses were not found when surgeons were confronted with a laboratory stressor (i.e., social stress test). Also, while the social stress test triggered cardiac autonomic and cortisol responses in both expert and senior surgeons, these responses did not correlate with those observed during surgery. These preliminary findings need to be replicated in larger samples, but suggest that individual differences in physiological responses to intraoperative stress (i) may be related to surgeons’ psychological characteristics, and (ii) may be typical of the surgical environment. Future research should seek to investigate the optimal level of cardiac autonomic and cortisol responses to intraoperative stress and how those surgeons most affected can be supported with stress management training programs. For example, in a randomized controlled study, surgeons who attended a stress management training showed higher HRV and increased coping skills during a simulated operation [37]. Further, qualitative analysis revealed improved technical skills, decision making, and confidence [37]. These interesting findings warrant further investigation on the effects of stress management training programs on cardiac autonomic and cortisol reactivity and recovery from real operations and their relationship with surgical performance.

### Limitations

The results of this exploratory study must be interpreted within the context of multiple potential confounders which are difficult to avoid in a real-life investigation of this kind, but should be considered and discussed. First, surgeons were monitored during operations of different nature and duration. However, all surgeons operated under similar work conditions (e.g., environmental characteristics of the operating room, number of assistant operators), complexities and complication rates were comparable, and the analysis of cardiac autonomic and cortisol responses was standardized to the first two hours of surgery. However, the length of surgery, entered as covariate in the statistical analysis of CAR measures, did not change the results. Also, it is unlikely that HR increases and HRV decreases during surgery were due to intraoperative posture and musculoskeletal strain and not to mental stress, as previously discussed [28]. Another possible confounder is the time of intraoperative assessment. All operations were scheduled in the morning, which is sub-optimal particularly for cortisol measures because it is known that cortisol levels peak shortly after awakening (i.e., CAR) and decline steadily into the early morning [3, 35]. Therefore, studies of cortisol stress reactivity frequently measure cortisol in the afternoon, on the premise that larger responses should occur against low afternoon baselines compared to high morning baselines. This may also explain why significant associations between psychometric characteristics and intraoperative stress responses were found for HR but not for cortisol reactivity.

## Conclusion

Two important gaps in the literature hamper our understanding of the possible relation between long-term exposure to intraoperative stress and high rates of burnout among surgeons. First, the extent to which intraoperative stress impacts on cardiac autonomic and HPA axis function in the aftermath of the surgery. Based on the results of this exploratory study, it is possible to put forward the hypothesis that intraoperative stress might have long-lasting effects on HPA axis function, warranting further studies aimed at (i) providing more robust evidence of a blunted CAR the day after surgery in larger samples and different medical specialties, and (ii) investigating whether this effect persists for an even longer period of time with an extension of cortisol sampling days. The second open issue is the extent to which individual psychobiological characteristics moderate the impact of intraoperative stress. The current results suggest that psychological characteristics might indeed be associated with intraoperative stress responses regardless of the surgeon’s level of experience and encourage the combined use of psychometric questionnaires and stress-related parameters (HR and HRV, cortisol) during and after real operations to deepen the study of this relationship.

We believe that these preliminary results will provide a springboard for large scale studies aimed at unveiling windows of vulnerability both at the personal (e.g., psychological features) and temporal (e.g., psychophysiological recovery from intraoperative stress) level, with the ultimate goal of improving surgical performance and patient safety on the one hand and preserving surgeons’ psychological well-being on the other hand. This could be achieved, for example, by testing the efficacy of stress managing interventions in more vulnerable surgeons and/or in the aftermath of a surgical procedure.

## Supplementary Information

**Additional file 1:** **Table S1.** Correlation coefficients (controlling for sex and experience) between cardiac autonomic and cortisol responses to social stress and surgery for the full sample of surgeons (*n*=16). **Table S2.** Correlation coefficients (controlling for sex and experience) between psychometric characteristic and heart rate variability and cortisol responses to surgery for the full sample of surgeons (*n*=16). **Table S3.** Correlation coefficients (controlling for sex and experience) between psychometric characteristic and cardiac autonomic and cortisol responses to social stress for the full sample of surgeons (*n*=16).

## Data Availability

The datasets used and/or analysed during the current study are available from the corresponding author on reasonable request.

## Abbreviations

CAR: Cortisol awakening response
CESD: Center for epidemiological studies depression scale
COPE: Coping orientation to problems experienced
DS-14: Type D personality scale
HPA: Hypothalamic–pituitary–adrenal
HR: Heart rate
HRV: Heart rate variability
PSS: Perceived stress scale
RMSSD: Root mean square of successive beat-to-beat interval differences
STAI: State-trait anxiety inventory

## References

[1] Alcantara JMA, Plaza-Florido A, Amaro-Gahete FJ, Acosta FM, Migueles JH, Molina-Garcia P (2020). Impact of Using Different Levels of Threshold-Based Artefact Correction on the Quantification of Heart Rate Variability in Three Independent Human Cohorts. J Clin Med.

[2] Arora S, Sevdalis N, Nestel D, Woloshynowych M, Darzi A, Kneebone R (2010). The impact of stress on surgical performance: a systematic review of the literature. Surgery.

[3] Azmi NASM, Juliana N, Azmani S, Effendy NM, Abu IF, Teng NIMF (2021). Cortisol on Circadian Rhythm and Its Effect on Cardiovascular System. Int J Environ Res Public Health.

[4] Balch CM, Shanafelt TD (2011). Burnout among surgeons: whether specialty makes a difference. Arch Surg.

[5] Balch CM, Shanafelt TD, Dyrbye L, Sloan JA, Russell TR, Bechamps GJ (2010). Surgeon distress as calibrated by hours worked and nights on call. J Am Coll Surg.

[6] Bohm B, Rotting N, Schwenk W, Grebe S, Mansmann U (2001). A prospective randomized trial on heart rate variability of the surgical team during laparoscopic and conventional sigmoid resection. Arch Surg.

[7] Carnevali L, Pattini E, Sgoifo A, Ottaviani C (2020). Effects of prefrontal transcranial direct current stimulation on autonomic and neuroendocrine responses to psychosocial stress in healthy humans. Stress.

[8] Chida Y, Steptoe A (2009). Cortisol awakening response and psychosocial factors: a systematic review and meta-analysis. Biol Psychol.

[9] Cohen S, Kamarck T, Mermelstein R (1983). A global measure of perceived stress. J Health Soc Behav.

[10] Demirtas Y, Tulmac M, Yavuzer R, Yalcin R, Ayhan S, Latifoglu O, et al. Plastic surgeon’s life: marvelous for mind, exhausting for body. Plastic Recons Sur. 2004;114(4):923–31. 10.1097/01.prs.0000133166.50279.7c. (discussion 932-923). 10.1097/01.prs.0000133166.50279.7c 15468400

[11] Denollet J (2005). DS14: standard assessment of negative affectivity, social inhibition, and Type D personality. Psychosom Med.

[12] Dimou FM, Eckelbarger D, Riall TS (2016). Surgeon Burnout: A Systematic Review. J Am Coll Surg.

[13] Dyrbye LN, West CP, Satele D, Boone S, Tan L, Sloan J, et al. Burnout among U.S. medical students, residents, and early career physicians relative to the general U.S. population. Acad Med : journal of the Association of American Medical Colleges. 2014;89(3):443–51. 10.1097/ACM.0000000000000134. 10.1097/ACM.0000000000000134 24448053

[14] Engelmann C, Schneider M, Kirschbaum C, Grote G, Dingemann J, Schoof S, Ure BM (2011). Effects of intraoperative breaks on mental and somatic operator fatigue: a randomized clinical trial. Surg Endosc.

[15] Erestam S, Bock D, Andersson AE, Haglind E, Park J, Angenete E (2021). The perceived benefit of intraoperative stress modifiers for surgeons: an experimental simulation study in volunteers. Patient Saf Surg.

[16] Fornari M, Carnevali L, Sgoifo A (2017). Single Osteopathic Manipulative Therapy Session Dampens Acute Autonomic and Neuroendocrine Responses to Mental Stress in Healthy Male Participants. J Am Osteo Assoc.

[17] Ganne C, Talkad SN, Srinivas D, Somanna S (2016). Ruptured blebs and racing hearts: autonomic cardiac changes in neurosurgeons during microsurgical clipping of aneurysms. Br J Neurosurg.

[18] Hansen TW, Thijs L, Boggia J, Li Y, Kikuya M, Bjorklund-Bodegard K (2008). Prognostic value of ambulatory heart rate revisited in 6928 subjects from 6 populations. Hypertension.

[19] Jarczok MN, Koenig J, Wittling A, Fischer JE, Thayer JF (2019). First Evaluation of an Index of Low Vagally-Mediated Heart Rate Variability as a Marker of Health Risks in Human Adults: Proof of Concept. J Clin Med.

[20] Jesuyajolu D, Nicholas A, Okeke C, Obi C, Aremu G, Obiekwe K, et al. Burnout among surgeons and surgical trainees: a systematic review and meta-analysis of the prevalence and associated factors. Surg Pract Sci. 2022;10(100094). 10.1016/j.sipas.2022.100094. 10.1016/j.sipas.2022.100094 PMC11749832 39845582

[21] Joseph B, Parvaneh S, Swartz T, Haider AA, Hassan A, Kulvatunyou N (2016). Stress among surgical attending physicians and trainees: A quantitative assessment during trauma activation and emergency surgeries. J Trauma Acute Care Surg.

[22] Kirschbaum C, Pirke KM, Hellhammer DH. The ’Trier Social Stress Test’–a tool for investigating psychobiological stress responses in a laboratory setting. Neuropsychobiology. 1993;28(1–2):76–81. 10.1159/000119004. 10.1159/000119004 8255414

[23] Kwon JW, Lee SB, Sung S, Park Y, Ha JW, Kim G (2021). Which Factors Affect the Stress of Intraoperative Orthopedic Surgeons by Using Electroencephalography Signals and Heart Rate Variability?. Sensors.

[24] Laborde S, Mosley E, Thayer JF (2017). Heart Rate Variability and Cardiac Vagal Tone in Psychophysiological Research - Recommendations for Experiment Planning, Data Analysis, and Data Reporting. Front Psychol.

[25] Oosterholt BG, Maes JHR, Van der Linden D, Verbraak M, Kompier MAJ (2015). Burnout and cortisol: evidence for a lower cortisol awakening response in both clinical and non-clinical burnout. J Psychosom Res.

[26] Pico Alfonso MA, Mastorci F, Ceresini G, Ceda G, Manghi M, Pino O, Troisi A, Sgoifo A (2007). Acute psychosocial challenge and cardiac autonomic response in women: the role of estrogens, corticosteroids, and behavioral coping styles. Psychoneuroendocrinology.

[27] Radloff LS (1977). The CES_D scale: A self-report depression scale for research in the general population. Appl Psychol Meas.

[28] Rieger A, Stoll R, Kreuzfeld S, Behrens K, Weippert M. Heart rate and heart rate variability as indirect markers of surgeons’ intraoperative stress. Int Arch Occup Environ Health. 2014;87(2):165–74. 10.1007/s00420-013-0847-z. 10.1007/s00420-013-0847-z 23370764

[29] Shanafelt TD, Balch CM, Bechamps G, Russell T, Dyrbye L, Satele D (2010). Burnout and medical errors among American surgeons. Ann Surg.

[30] Sica C, Magni C, Ghisi M, Altoè G, Sighinolfi C, Chiri LR, et al. Coping Orientation to Problems Experienced-Nuova Versione Italiana (COPE-NVI): uno strumento per la misura degli stili di coping. Psicoterapia cognitiva e comportamentale. 2008;14(27).

[31] Spielberger CD, Gorsuch RL, Lushene R, Vagg PR, Jacobs GA (1983). Manual for the state-trait inventory.

[32] Tarvainen MP, Niskanen JP, Lipponen JA, Ranta-Aho PO, Karjalainen PA (2014). Kubios HRV–heart rate variability analysis software. Comput Methods Programs Biomed.

[33] The AF, Reijmerink I, van der Laan M, Cnossen F (2020). Heart rate variability as a measure of mental stress in surgery: a systematic review. Int Arch Occup Environ Health.

[34] Weenk M, Alken APB, Engelen L, Bredie SJH, van de Belt TH, van Goor H (2018). Stress measurement in surgeons and residents using a smart patch. Am J Surg.

[35] Weitzman ED, Fukushima D, Nogeire C, Roffwarg H, Gallagher TF, Hellman L (1971). Twenty-four hour pattern of the episodic secretion of cortisol in normal subjects. J Clin Endocrinol Metab.

[36] Wetzel CM, Black SA, Hanna GB, Athanasiou T, Kneebone RL, Nestel D (2010). The effects of stress and coping on surgical performance during simulations. Ann Surg.

[37] Wetzel CM, George A, Hanna GB, Athanasiou T, Black SA, Kneebone RL (2011). Stress management training for surgeons-a randomized, controlled, intervention study. Ann Surg.

[38] Wetzel CM, Kneebone RL, Woloshynowych M, Nestel D, Moorthy K, Kidd J (2006). The effects of stress on surgical performance. Am J Surg.

