# Supplementary material for: Cardiac autonomic and cortisol stress responses to real operations in surgeons: relationship with individual psychobiological characteristics and experience
Source: Biopsychosoc Med. 2023 Feb 21;17:5. doi: 10.1186/s13030-023-00266-5 (PMC9942282; doi:10.1186/s13030-023-00266-5)
Supplement: Supplementary file 1 — Additional file 1: Table S1. Correlation coefficients (controlling for sex and experience) between cardiac autonomic and cortisol responses to social stress and surgery for the full sample of surgeons (n=16). Table S2. Correlation coefficients (controlling for sex and experience) between psychometric characteristic and heart rate variability and cortisol responses to surgery for the full sample of surgeons (n=16). Table S3. Correlation coefficients (controlling for sex and experience) between psychometric characteristic and cardiac autonomic and cortisol responses to social stress for the full sample of surgeons (n=16). [file 13030_2023_266_MOESM1_ESM.docx]

**Table S1**. Correlation coefficients (controlling for sex and experience) between cardiac autonomic and cortisol responses to social stress and surgery for the full sample of surgeons (n=16).

|  | 1 | 2 | 3 | 4 | 5 | 6 |
| --- | --- | --- | --- | --- | --- | --- |
| 1. delta HR social stress | - |  |  |  |  |  |
| 2. delta RMSSD social stress | -.307 | - |  |  |  |  |
| 3. delta CORT social stress | .555* | .046 | - |  |  |  |
| 4. delta HR surgery | -.443 | .284 | -.323 | - |  |  |
| 5. delta RMSSD surgery | -.023 | .265 | .309 |  | - |  |
| 6. delta CORT surgery | -.513 | -.258 | -.264 | -.073 | -.229 | - |

*Note.* Abbreviations: HR = heart rate; RMSSD = root mean square of successive beat-to-beat interval differences; CORT = cortisol. * = p < .05.

**Table S2**. Correlation coefficients (controlling for sex and experience) between psychometric characteristic and heart rate variability and cortisol responses to surgery for the full sample of surgeons (n=16).

|  | 1 | 2 | 3 | 4 | 5 | 6 | 7 |
| --- | --- | --- | --- | --- | --- | --- | --- |
| 1. DS14 (NA) | - |  |  |  |  |  |  |
| 2. STAI-T | .738* | - |  |  |  |  |  |
| 3. STAI-S | .422 | .248 | - |  |  |  |  |
| 4. CESD | .509 | .798* | .430 | - |  |  |  |
| 5. PSS | .471 | .509 | .229 | .618* | - |  |  |
| 6. delta RMSSD | -.160 | -.220 | -.143 | -.124 | -.312 | - |  |
| 7. delta cortisol | -.423 | -.340 | -.021 | -.180 | -.018 | -.229 | - |

*Note.* Abbreviations: DS-14 (NA) = Type D Personality Scale, negative affectivity subscale; STAI-T = State-Trait Anxiety Inventory, Trait version; STAI-S = State-Trait Anxiety Inventory, State version; CESD = Center for Epidemiological Studies Depression Scale; PSS = Perceived Stress Scale; RMSSD = root mean square of successive beat-to-beat interval differences; * = p < .05.

**Table S3**. Correlation coefficients (controlling for sex and experience) between psychometric characteristic and cardiac autonomic and cortisol responses to social stress for the full sample of surgeons (n=16).

|  | 1 | 2 | 3 | 4 | 5 | 6 | 7 | 8 |
| --- | --- | --- | --- | --- | --- | --- | --- | --- |
| 1. DS14 (NA) | - |  |  |  |  |  |  |  |
| 2. STAI-T | .738* | - |  |  |  |  |  |  |
| 3. STAI-S | .255 | .211 | - |  |  |  |  |  |
| 4. CESD | .532* | .718* | .447 | - |  |  |  |  |
| 5. PSS | .348 | .412 | .670* | .553* | - |  |  |  |
| 6. delta HR | -.162 | -.263 | .208 | .140 | .159 | - |  |  |
| 7. delta RMSSD | -.246 | .407 | .219 | .336 | .106 | -.307 | - |  |
| 8. delta cortisol | -.070 | -.267 | .158 | -.147 | .010 | .555* | .046 | - |

*Note.* Abbreviations: DS-14 (NA) = Type D Personality Scale, negative affectivity subscale; STAI-T = State-Trait Anxiety Inventory, Trait version; STAI-S = State-Trait Anxiety Inventory, State version; CESD = Center for Epidemiological Studies Depression Scale; PSS = Perceived Stress Scale; HR = heart rate; RMSSD = root mean square of successive beat-to-beat interval differences; * = p < .05.
